# High grade isthmic spondylolisthesis; can reduction always re-align the unbalanced pelvis?

**DOI:** 10.1186/s12891-019-2865-9

**Published:** 2019-10-29

**Authors:** Konstantinos Martikos, Tiziana Greggi, Cesare Faldini

**Affiliations:** Istituto Ortopedico Rizzoli, Via Pupilli 1, 40136 Bologna, Italy

**Keywords:** Isthmic spondylolisthesis, Pelvic balance, Interbody fusion, Meyerding classification

## Abstract

**Background:**

Recently, various studies have reported the importance of distinguishing between balanced and unbalanced SL, sustaining the importance of SL reduction in unbalanced cases. In this study we present our experience in the treatment of isthmic spondylolisthesis in young patients, observing the correlation between SL reduction and sagittal correlation between spine and pelvis.

**Methods:**

This is a retrospective study of a series of patients treated surgically for isthmic spondylolisthesis. Inclusion criteria were L5-S1 isthmic spondylolisthesis of III° or IV°, pediatric age, clinical and radiographic follow up of at least 1 year. Radiographic evaluation included the following elements: grade and percentage of listhesis (%L), lumbar lordosis (LL), lumbar-sacral angle (LSA), pelvic incidence (PI), sacral slope (SS), pelvic tilt (PT) distinguishing between “balanced” and “unbalanced” patients. Radiographic values were confronted by using Student’s t- test, obtaining a statistically significant difference for values inferior to 0,05.

**Results:**

Based on inclusion criteria, 28 patients were selected for our retrospective analysis, 19 female and 9 male. Mean age at surgery was 15,6 years. Mean follow up was 3 years and 3 months (min. 1 year – max 6 years and 7 months). Spondylolisthesis reduction was statistically significant both in balanced and in unbalanced patients, but pelvic incidence values did not improve significantly. We observed fewer mechanical complications in patients treated with interbody support.

**Conclusion:**

In our study, differences between pre-op and post-op spinopelvic alignment values were not statistically significant, even though spondylolisthesis reduction was statistically significant in all cases. Our study could be considered an initial attempt to correlate spinopelvic changes to spondylolisthesis reduction in a progressive manner, and possibly in the future, generate threshold values of reduction for ideal spinopelvic alignment in every different patient.

## Background

Isthmic spondylolisthesis (SL) represents a sagittal deformity of the spine, thus influencing both sagittal alignment of the pelvis both from the clinical and radiographic point of view. Therefore, the objective of surgical treatment is not only the stabilization of the instable segment but also reduction of the slipped vertebra over S1. Recently, various studies have reported the importance of distinguishing between balanced and unbalanced SL, sustaining the importance of SL reduction in unbalanced cases. More precisely, various studies in the recent years sustain that reducing a high-grade spondylolisthesis may balance un unbalanced pelvis, without focusing on results in cases of incomplete spondylolisthesis reduction.

In this study we present our experience in the treatment of isthmic spondylolisthesis in young patients, observing the correlation between SL reduction and sagittal pelvic alignment in terms of pelvic tilt, sacral slope and lumbar-sacral angle, with a critical view on pelvic balance especially in cases of satisfactory but not complete spondylolisthesis reduction.

## Methods

This is a retrospective study of a series of patients treated surgically for isthmic spondylolisthesis in a single spinal deformities division between 2003 and 2012. Inclusion criteria were the following: patients affected by L5-S1 isthmic spondylolisthesis of III o IV grade according to Meyerding, age between 10 and 18 years, posterior pedicle screw instrumentation, clinical and radiographic follow up of at least 1 year.

Radiographic evaluation included the following elements: grade and percentage of listhesis (%L) according the Mayerding scale, lumbar lordosis (LL, angle between L1 and S1 upper endplates), lumbar-sacral angle (LSA, angle between L5 lower endplate and S1 upper endplate), pelvic incidence (PI, angle between the line perpendicular to the sacral plate at its midpoint and the line connecting this point to the femoral heads axis), sacral slope (SS, angle between S1 upper endplate and the horizontal plane), pelvic tilt (PT, angle between a vertical line and the line joining the middle of the sacral plate and the axis of the femoral heads).

Our series was analyzed both in its entirety as well as divided in two groups of “balanced” and “unbalanced” patients. The concepts of balanced and unbalanced spondylolisthesis were based on previous reports of Hresko at al [1]., that distinguish high SS-low PT as balanced ones and low SS-high PT as unbalanced ones, with the latter ones presenting retroverted pelvis and vertical sacrum.

All radiographic measurements were performed upon pre-operatory, post-operatory and last follow up x-rays by the author of the study. Radiographic values were confronted by using Student’s t- test, obtaining a statistically significant difference for values inferior to 0,05.

Based on inclusion criteria, 28 patients were selected for our retrospective analysis, 19 female (67.9%) and 9 male (32.1%) (Table 1). Mean age at surgery was 15,6 years (min. 10 - max 18 years). All patients had spondylolisthesis superior to 50%, being therefore classified as grade III or IV according to Meyerding. Mean slippage percentage was 81,04% (min 57% - max 100%). Mean follow up was 3 years and 3 months (min. 1 year – max 6 years and 7 months).

**Table 1:** Mean demographic values of patient population

Age	**15.61** years (*10–18*)
Sex	**19 F** (67,9%), **9 M** (32,1%)
Grade III° SL	**12** (42,8%)
Grade IV° SL	**16** (57,2%)
Posterior instrumentation with PLIF	**4** (14,3%)
Posterior instrumentation with TLIF	**6** (21,4%)
Only anterior stabilization	**1** (3,5%)
Posterior and anterior stabilization	**4** (14,3%)
*-Anterior L5-S1 screw*	***2*** *(7,1%)*
*-ALIF*	***1*** *(3,5%)*
*-Autologous bone only*	***1*** *(3,5%)*
Sonly posterior pedicle screw instrumentation	**15** (53,5%)
L5-S1 fusion	**19**
L4-S1 fusion	**9**

Baseline surgical treatment was posterolateral instrumented fusion in all patients. Posterior arch of L5 was removed in all cases prior to reduction maneuvers for an optimal radicular release. Spondylolisthesis was reduced to some point, although not totally in all patients. In 19 cases, instrumentation was limited to L5, in 9 patients it was extended to L4. In 15 patients interbody support was associated to posterior instrumentation:

- Posterolateral lumbar interbody fusion (PLIF) in 4 cases (cages in peek material in 2 cases, titanium in 2 cases).
- Transforaminal lumbar interbody fusion (TLIF) in 6 cases (cages in peek material in 4 cases, titanium in 2 cases).
- Posterior L5-S1 transdiscal screw in 1 case.
- Anterior fusion in 4 cases, performed at mean 4,2 months after initial posterior fusion (min 1 – max 8 months). Of these patients, two were treated with a single L5-S1 screw, one was treated with an anterior L5-S1 transdiscal screw and one was treated with autogenous interbody bone graft.

Our mean pre-operatory spinal-pelvic values are consistent with previously reported values in high grade isthmic spondylolisthesis, mainly characterized by high PI and PT [2–4]:

PI: 72.78° (normal value 48.4°).

PT: 28° (normal value 7.2)°.

SS: 43.89° (normal value 41.2°).

LL: 57.81° (normal value 48.5°).

## Results

Spinal and pelvic radiographic measurements of the entire series are reported in Table 2. Considering the whole series (without distinguishing between balanced and unbalanced patients), we found a statistically significant improvement in %L (*p* = 0,0000017) and LSA (*p* = 0,003) between pre-operative and post-operative values. No statistically significant differences were found regarding LL, SS, PT and PI between pre and post-operative values. Although post-operative values of SS and PT showed a trend towards normal population values when compared to preoperative values (SS increased and PT declined) the difference was not statistically significant. Values remained stable at final FU, with no statistically significant differences between post-op and final FU.

**Table 2:** Mean pre-op, post-op and final FU values, in all patients

X-Ray index	Pre-op	Post-op	FU
Listhesis percentage	81% *(57–141)*	43,6%*(0–149)*	58,8%*(5–154)*
Lumbar-sacral angle (LSA)	−19,5° *(− 47 to + 17)*	−6,6° *(− 58 to + 15)*	−12,3° *(− 47 to + 15)*
Lumbar lordosis (LL)	57,8° *(29–72)*	56,4° *(40–72)*	55,1° *(38–71)*
Sacral slope (SS)	43,9° (20–59)	45,9° (18–57)	43,9° (19–60)
Pelvic tilt (PT)	28,2° (17–41)	25,6° (11–45)	28,8° (14–49)
Pelvic incidence (PI)	72,8° (49–88)	72,1° (51–88)	73,1° (41–89)

The series was afterwards split in two groups, balanced and unbalanced patients, based on pre-operatory pelvic values as defined in the Hresko et al. paper [1]: balanced patients group with high SS and low PT (11 patients) and unbalanced patients group with low SS and high PT (17 patients). Results were similar to the entire series evaluation. Neither group showed statistically significant differences in pelvic parameters between pre-op ad post-op values. Although there was a statistically significant listhesis reduction as well as LSA improvement, pelvic parameters (SS, PT, LL) did not seem to show a statistically significant improvement in neither balanced or unbalanced group of patients (Tables 3 and 4). Lastly, our case series was divided into two groups based on the presence or not of interbody support at first surgical procedure (PLIF, TLIF, ALIF, transdiscal screw) and sagittal spinal-pelvic parameters were calculated before and after surgery (Tables 5 and 6). Both posterior + interbody instrumented cases as well as those with only posterior instrumentation showed a statistically significant %L reduction: 78% mean pre-op vs 23,5% mean post-op, *p* = 0,0001 in posterior + interbody instrumented cases 83,2% pre-op vs 56% post-op, *p* = 0,00001 in posterior only instrumented cases without interbody support.

**Table 3:** Mean pre-op, postop and FU values of “balanced” group patients

X-Ray index	Pre-op	Post-op	FU
Listhesis percentage	79,6% *(57–112)*	41,2% *(0–119)*	52,9% *(5–116)*
Lumbar-sacral angle (LSA)	−15,5° *(da − 33 a + 17)*	− 3° *(da − 35 a + 15)*	− 7,4° *(da − 45 a + 15)*
Lumbar lordosis (LL)	63,73° *(56–72)*	60,9° *(51–72)*	61,2° *(55–71)*
Sacral slope (SS)	50° (45–59)	48,9° (43–59)	47,5° (31–59)
Pelvic tilt (PT)	27° (17–36)	24,4° (11–40)	26,9° (14–39)
Pelvic incidence (PI)	74,5° (64–86)	74° (60–88)	74,9° (60–88)

**Table 4:** Mean pre-op, postop and FU values of “unbalanced” group patients

X-Ray index	Pre-op	Post-op	FU
Listhesis percentage	81% *(63–141)*	45,6% *(4–149)*	62,5% *(19–154)*
Lumbar-sacral angle (LSA)	−22° *(− 58 to + 15)*	−9,1° *(− 58 to + 15)*	− 15,4° *(− 47 to + 11)*
Lumbar lordosis (LL)	54,53° *(68–29)*	53° *(40–63)*	50,7° *(38–65)*
Sacral slope (SS)	40° (20–58)	44° (19–53)	41,6° (19–60)
Pelvic tilt (PT)	29,4° (17–41)	24,1° (13–45)	30,8° (15–49)
Pelvic incidence (PI)	71,9° (49–88)	71,6° (51–87)	72,7° (41–89)

**Table 5:** Mean pre-op, post-op and final FU values in patients treated with interbody support

X-Ray index	Pre-op	Post-op	FU
Listhesis percentage	77,8% *(57–98)*	23.5% *(1–71)*	29.5% *(5–116)*
Lumbar-sacral angle (LSA)	−15,8° *(− 3 to − 31)*	4.7° *(− 7 to + 15)*	0.6° *(− 45 to + 15)*
Lumbar lordosis (LL)	60° *(37–72)*	58,3° *(40–72)*	56.7° *(40–66)*
Sacral slope (SS)	43.4° (20–55)	47.2° (19–59)	43.9° (19–59)
Pelvic tilt (PT)	28.3° (17–36)	21.6° (11–38)	24.4° (14–39)
Pelvic incidence (PI)	70,5° (56–86)	69.5° (51–88)	69.8° (51–88)

**Table 6:** Mean pre-op, post-op and final FU values in patients treated without interbody support

X-Ray index	Pre-op	Post-op	FU
Listhesis percentage	83,2% *(63–141)*	56,6% *(4–149)*	75,3% *(30–154)*
Lumbar-sacral angle (LSA)	− 22° (*−47 to + 17)*	− 13° *(− 58 to + 15)*	−19,5° *(− 47 to + 15)*
Lumbar lordosis (LL)	56,2° *(29–71)*	55,1° *(43–68)*	54,2° *(38–71)*
Sacral slope (SS)	44,2° *(26–59)*	45° *(37–56)*	43,9° *(22–56)*
Pelvic tilt (PT)	28,6° *(17–41)*	28,1° *(13–45)*	31,2° *(16–49)*
Pelvic incidence (PI)	72,9° (49–88)	73,3° (58–85)	73,6° (41–89)

At final FU we observed that the interbody support group maintained listhesis reduction while the posterior only instrumented patients presented a loss of listhesis reduction when compared to immediate post-operative results. This difference between the two groups was statistically significant (p = 0,0001). %L: 23.5% post-op vs 29.5% at final FU in the interbody support patients and 56.65% post-op vs 75.31% at final FU in patients without interbody support.

Patients treated with interbody support showed statistically better LSA improvement when compared to patients treated without interbody support: LSA improvement of 25% in patients treated with interbody support (*p* < 0.0001) vs 8.1% (*p* = 0.17) in patients treated without interbody support. LSA: 4.7° post-op vs 0.6° at final FU in interbody support patients and − 13.9° post-op vs − 19.5° at final FU in patients without interbody fusion. The remaining sagittal spinal-pelvic parameters (LL, SS, PT and PI) remained statistically similar, both within each group before and after surgical treatment, and between the two groups when compared to each other.

Mechanical complications with loss of correction were observed in 4 patients (14.2%). Revision surgery was performed in each case, with the objective to obtain satisfactory listhesis stabilization, not necessarily reduction. All mechanical complications occurred in patients treated without interbody support at primal surgery. Therefore, mechanical complications incidence in patients treated without interbody support at primal surgery was 24%. No mechanical complications were observed in patients treated with interbody support. Difference in mechanical complications was therefore statistically significant between these two groups of patients (*p* < 0,00001).

We observed 2 cases of bilateral sacral screw breakage (7.1%) that occurred after a mean 4,5 months (2 and 7 months respectively). Both patients were initially treated with posterior instrumented arthrodesis L4-S1 without any interbody support. In both cases revision surgery was performed by removing the broken screws and replacing them with greater diameter ones. In one case, anterior instrumentation with two L5- S1 screws was performed.

L5 screw pull out was observed in 2 patients (7.1%), unilateral in 1 case and bilateral in the other, after a mean 4,5 months from initial surgery (3 and 6 months respectively). Both cases underwent revision surgery by substituting mobilized screws with greater diameter ones. In one patient instrumentation was was extended to L4 and anterior stabilization with AxiaLIF was performed. In one patient treated with TLIF cages, subsidence was observed (3.5%) 1 month after surgery, without symptoms and without listhesis progression.

No infections were registered.

Two patients experienced neurologic symptoms immediately after surgery. Both patients had unilateral L5 nerve root incomplete motor deficit that recovered completely after a mean two months period.

## Discussion

The limits of our study are its retrospective design, the limited number of patients and the diversity in surgical technique applied through a 10-year period by spine surgeons of our Institute. On the other hand, we should consider that high grade isthmic spondylolisthesis is a relatively rare disease and surgical techniques have drastically changed over time.

Patients treated with interbody support showed statistically better LSA improvement.

when compared to patients treated without interbody support. At final follow up, patients treated with interbody support showed a statistically significant better stability both in terms of LSA and %L.

Mechanical complications occurred in 4 patients all of whom were treated with posterior only fusion without interbody support, confirming therefore the utility of interbody support. All mechanical complications were treated with revision surgery, obtaining segmental stability at final FU in all patients.

We did not have any major or permanent neurologic deficits or infections.

The most important outcome of this study is represented by considerations upon pelvic balance. Our study confirms literature data [5], regarding elevated spinalpelvic radiographic parameters in pediatric patients affected by high grade isthmic spondylolisthesis. Our mean pre-operatory spinal-pelvic values are consistent with previously reported values in high grade isthmic spondylolisthesis, mainly characterized by high PI and PT.

Our study data differs from those of other studies when we take under consideration spinopelvic radiographic changes that occur after spondylolisthesis surgical treatment. Recent papers regarding surgical treatment of high grade isthmic spondylolisthesis, sustain that listhesis reduction in patients with unbalanced pelvis can restore sagittal pelvic alignment and therefore adapt spinopelvic radiographic parameters towards normal values [1, 6, 7]. In our study, we did observe spinopelvic alignment improvement but differences between pre-op and post-op values were not statistically significant. Even when we considered only “unbalanced” patients according do the Hresco et al. nomogram [1], we did not observe statistically significant differences in spinopelvic alignment parameters between preop and postop, although spondylolisthesis reduction was statistically significant, in terms of both %L and LSA. Even when we divided patients according to the presence of interbody support, neither group of patients showed a statistically significant difference between pre and post-op.

The 2009 paper from Hresko et al. [8] sustains similar results to our data, sustaining that “in unbalanced spondylolisthesis, pelvic sagittal balance improved in 75% of patients with reduction, but did not correlate to the amount of reduction of spondylolisthesis”.

## Conclusions

According to our data, spondylolisthesis reduction may not provide ideal pelvic alignment, in other words incomplete spondylolisthesis reduction in an initially unbalanced pelvis may still provide unbalanced pelvis after surgery. Presenting a case series of continuous patients affected by high grade isthmic spondylolisthesis that did not have a total spondylolisthesis reduction in each and every case represents a realistic approach to this pathology, in contrast to other recent studies reporting total listhesis reduction in all patients correlated to ideal pelvic sagittal restoration in all patients. This study may therefore contribute to explore the vast amount of information that extends between a high grade listhesis and its perfect reduction. Our study could be considered an initial attempt to correlate spinopelvic changes to spondylolisthesis reduction in a progressive manner, and possibly in the future, generate threshold values of reduction for ideal spinopelvic alignment in every different patient.

## Data Availability

The datasets used and/or analyzed during the current study are available from the corresponding author on reasonable request.

## Abbreviations

FU: Follow up
L: Listhesis (or slippage)
LL: Lumbar lordosis
LSA: Lumbar-sacral angle
PI: Pelvic incidence
PLIF: Posterior lumbar interbody fusion
Post-op: Post-operatory
Pre-op: Pre-operatory
PT: Pelvic tilt
SS: Sacral slope
TLIF: Transforaminal lumbar interbody fusion
